# Identification of risk factors for residual cholesteatoma in children and adults: a retrospective study on 110 cases of revision surgery^☆^

**DOI:** 10.1016/j.bjorl.2018.11.004

**Published:** 2018-12-31

**Authors:** Veronika Volgger, Göran Lindeskog, Eike Krause, Florian Schrötzlmair

**Affiliations:** Klinikum der Universität München, Department of Otorhinolaryngology, Head & Neck Surgery, München, Germany

**Keywords:** Cholesteatoma, Canal-wall-up, Canal-wall-down, Residual cholesteatoma, Colesteatoma, Canal *wall-up*, Canal *wall-down*, Colesteatoma residual

## Abstract

**Introduction:**

Residual disease after cholesteatoma removal is still a challenge for the otorhinolaryngologist. Scheduled “second-look” surgery and, more recently, radiological screenings are used to identify residual cholesteatoma as early as possible. However, these procedures are cost-intensive and are accompanied by discomfort and risks for the patient.

**Objective:**

To identify anamnestic, clinical, and surgery-related risk factors for residual cholesteatoma.

**Methods:**

The charts of 108 patients, including children as well as adults, having undergone a second-look or revision surgery after initial cholesteatoma removal at a tertiary referral hospital, were analyzed retrospectively.

**Results:**

Gender, age, mastoid pneumatization, prior ventilation tube insertion, congenital cholesteatoma, erosion of ossicles, atticotomy, resection of chorda tympani, different reconstruction materials, and postoperative otorrhea did not emerge as statistically significant risk factors for residual disease. However, prior adenoid removal, cholesteatoma growth to the sinus tympani and to the antrum and mastoid, canal-wall-up 2 ways approach, and postoperative retraction and perforation were associated with a statistically higher rate of residual disease. A type A tympanogram as well as canal-wall-down plus reconstruction 2 ways approach for extended epitympanic and for extended epitympanic and mesotympanic cholesteatomas were associated with statistically lower rates of residual disease. A score including the postoperative retraction or perforation of the tympanic membrane, the quality of the postoperative tympanogram and the intraoperative extension of the cholesteatoma to the sinus tympani and/or the antrum was elaborated and proved to be suitable for predicting residual cholesteatoma with acceptable sensitivity and high specificity.

**Conclusion:**

Cholesteatoma extension to the sinus tympani, antrum and mastoid makes a residual disease more likely. The canal-wall-down plus reconstruction 2 ways approach seems safe with similar rates of residual cholesteatoma and without the known disadvantages of canal-wall-down surgery. The described score can be useful for identifying patients who need a postoperative radiological control and a second-look surgery.

## Resumo

**Introdução:**

A doença residual após a remoção do colesteatoma ainda é um desafio para o otorrinolaringologista. A cirurgia revisional programada e, mais recentemente, exames radiológicos são usados para identificar o colesteatoma residual o mais precocemente possível. Entretanto, esses procedimentos são dispendiosos e acompanhados de desconforto e riscos para o paciente.

**Objetivo:**

Identificar fatores de risco anamnésicos, clínicos e relacionados à cirurgia para o colesteatoma residual.

**Método:**

Foram analisados retrospectivamente os prontuários de 108 pacientes, crianças e adultos, que passaram por revisão cirúrgica após a remoção inicial do colesteatoma em um hospital terciário de referência.

**Resultados:**

Sexo, idade, pneumatização da mastoide, inserção anterior de tubo de ventilação, colesteatoma congênito, erosão dos ossículos, aticotomia, ressecção da corda do tímpano, diferentes materiais de reconstrução e otorreia pós-operatória não se mostraram fatores de risco estatisticamente significantes para a ocorrência de doença residual. Entretanto, remoção prévia da adenoide, crescimento do colesteatoma para o interior do seio timpânico e para o antro e a mastoide, abordagem de duas vias com canal *wall-up* e retração e perfuração pós-operatórias foram associados a uma taxa estatisticamente maior de doença residual. Um timpanograma tipo A, assim como a reconstrução de duas vias com a abordagem canal *wall-down* para colesteatomas com extensão para o recesso epitimpânico e/ou extensão epitimpânica e mesotimpânica, foram associados com taxas estatisticamente menores da doença residual. Um escore, que incluiu a retração ou perfuração pós-operatória da membrana timpânica, a qualidade do timpanograma pós-operatório e a extensão intraoperatória do colesteatoma para o seio timpânico e/ou antro, foi elaborado e se mostrou adequado para predizer colesteatoma residual com sensibilidade aceitável e alta especificidade.

**Conclusão:**

A extensão do colesteatoma para o seio timpânico, antro e mastoide torna a doença residual mais provável. A abordagem do tipo canal *wall-down* mais a reconstrução de 2 vias parecem seguras com taxas semelhantes de colesteatoma residual e sem as desvantagens conhecidas da cirurgia do tipo canal *wall-down*. O escore descrito pode ser útil para identificar pacientes que necessitam de controle radiológico pós-operatório e cirurgia revisional.

## Introduction

Cholesteatoma is a common health problem in Europe with a yearly incidence of about 3/100,000 in children and 9.2/100,000 in adults. Most cases are acquired, while only 4% of cholesteatomas in children are congenital.^1^, ^2^ Acquired cholesteatoma results from a retraction pocket, from invasion of squamous cell epithelium from the outer ear canal^3^ or as a consequence of prior surgery or infection.^4^ Cholesteatoma generally requires complete surgical removal which is done by Canal-Wall-Up (CWU) or Canal-Wall-Down (CWD) surgery. In CWD procedures the posterior wall of the auditory canal is partially removed, usually allowing a good visualization of the middle ear. Furthermore, the detection of recurrences in the follow-up period should be facilitated. However, CWD surgery often results in reduced earwax and dander cleaning and subsequent recurrent cavity infections with otorrhea.^5^ In CWU procedures the posterior auditory canal wall remains intact, on the one hand accelerating postoperative healing and reducing the need of frequent cleaning of the outer ear canal. On the other hand, the intraoperative visualization for complete cholesteatoma removal might be limited and it might be more difficult to identify residual disease.^5^ CWD surgery can be combined with a reconstruction of the previously removed parts of the posterior auditory canal wall. With this approach a good intraoperative overview should be guaranteed and the postoperative problems from a classic CWD surgery should be avoided.

Cholesteatomas bear a relevant risk of residual disease. In the literature rates of residual cholesteatoma considerably vary and range from 12.3% to 67.0%, mainly due to inconsistent study designs.^6^, ^7^, ^8^, ^9^, ^10^, ^11^, ^12^, ^13^, ^14^ Despite the variety of trials, various risk factors for residual cholesteatoma seem to have been identified, including ossicular erosion,^6^, ^10^, ^11^, ^12^, ^15^ growth toward the sinus tympani^10^, ^11^, ^13^ or the posterosuperior region,^6^, ^8^ extended size of cholesteatoma,^11^, ^15^, ^16^ inexperience of the surgeon^11^ and cholesteatoma in children.^13^, ^17^ Even though CWD procedures seem to have a lower recurrence rate Mishiro et al. proposed an individual decision depending on patient and cholesteatoma extension whether to go with CWD, CWU or CWD with reconstruction of the posterior auditory canal wall.^18^ Patients with a high risk of residual disease usually undergo a second-look surgery. However, the recommendation for this operation some months after cholesteatoma removal remains a matter of controversy. On the one hand, it offers the possibility of an early detection of residual disease, while on the other hand, it bears the risk that patients are exposed to the danger of a second surgery and general anesthesia for no benefit. In this context, great efforts have been made in recent years to improve sensitivity and specificity of radiological follow-up examinations, e.g., diffusion-weighted MRI scans.^19^ However, these radiological scans are relatively cost-intensive. The recommendation of a postoperative radiological control or a second-look operation is still based on the surgeon's impression during cholesteatoma removal as to date there exist no guidelines. To this end, we wanted to identify anamnestic, clinical, and surgery-related risk factors for residual cholesteatoma by a follow-up investigation of all patients having undergone a second-look operation or revision tympanoplasty after initial cholesteatoma removal in our department. Including both children and adults, our study design also allows the comparison of residual cholesteatoma rates in patients of different age.

## Methods

### Patients

Between 01/01/1995 and 12/31/2011 268 patients underwent an initial cholesteatoma removal in a single tertiary referral center. 108 of these patients underwent a second surgery, two of them on both ears. In those two patients the ears were considered separately, leading to 110 cases of either scheduled second-look operations (*n* = 61) or unplanned revision surgeries (*n* = 49), due to clinical suspicion for cholesteatoma. The charts of all patients with a second surgery were analyzed in order to identify risk factors for residual cholesteatoma. The 158 patients who had been operated only once were not further analyzed. The inclusion was independent from the patient's age and type of cholesteatoma (congenital, acquired). Exclusion criterion was a simultaneous additional surgery, such as adenoidectomy or tonsillectomy. All patients were operated by three experienced surgeons, exclusively doing ear surgery.

The ratio of male to female patients was around 1.3:1. The mean age at the second-look operation was 30.12 years (SD = 20.26 years, range: 5–78 years). Table 1 gives an overview of all patients investigated. It shows the number of patients who received a recommendation for a second-look operation and how many of these recommended operations really took place. Furthermore, the cases of unscheduled revision surgery were included. All cases with cholesteatoma tissue in second surgery were considered as cases of residual cholesteatoma.

**Table 1:** Rates of residual cholesteatoma depending on the recommendation of a second-look surgery.

Second-look	Total number of patients (268)	Patients whose second-look surgery took place, 110 (41.0%)	Residual cholesteatoma, 63 (57.3%)	No residual cholesteatoma, 47 (42.7%)
Surgery recommended	92 (34.3%)	61 (66.3%)	29 (47.5%)	32 (52.5%)
Surgery not recommended	176 (65.7%)	49 (27.8%)	34 (69.4%)	15 (30.6%)

The column “total number of patients” shows the absolute and relative number of patients treated for cholesteatoma at our department between 01/01/1995 and 12/31/2011. The column “patients whose second-look surgery took place” shows the absolute and relative number of patients undergoing a second-look surgery, taking into account if this operation had been recommended or not, while the last two columns show the absolute and relative number of patients having or not having a residual cholesteatoma in second-look surgery.

### Statistical analysis

All data were pseudonymized and recorded in a Microsoft Excel sheet. The statistical analysis was performed using IBM SPSS Statistics 20 for Windows. The various collected parameters were listed in a fourfold table to identify risk factors. The distribution of the various parameters throughout the group with and without residual disease was compared using the Chi^2^-test. In cases where only a small number of data could be collected for a certain parameter, the Fisher's exact test was used instead. The significance level was stated as *p* ≤ 0.05. For *p* ≤ 0.01 the result was considered as statistically highly significant. For fourfold tables the Odds Ratio (OR) was calculated to compare the risk for a residual cholesteatoma in presence or absence of a certain parameter. Parameters that were identified as statistically significant regarding residual disease in a univariate analysis were further analyzed in a multivariate analysis. Using the logistical regression, interdependencies between the tested variable and other variables were revealed.

## Results

### Preoperative risk factors before initial cholesteatoma removal

Neither gender (*p* = 0.294) nor age (*p* = 0.841) was identified as statistically significant risk factors for residual cholesteatoma. The rate of residual cholesteatoma for patients <18 years (*n* = 45) and patients ≥18 years (*n* = 65) was comparable (60.0% in children vs. 55.4% in adults). There was no statistically significant difference (*p* = 0.630).

Prior insertion of ventilation tubes (*n* = 33 of 108 patients) was not associated with a higher risk of residual disease (*p* = 0.668), whereas a prior removal of adenoids was a statistically significant risk factor (*p* = 0.019). In 73.0% of patients (*n* = 27/37 patients) having had a prior adenoidectomy residual disease was found in the second-look surgery while only 47.9% of patients (*n* = 34/71 patients) without adenoidectomy suffered from residual cholesteatoma (OR = 2.94). When compared with other parameters in a multivariate analysis (retraction, perforation), prior adenoid removal was identified as an independent risk factor.

### Intraoperative risk factors during initial cholesteatoma removal

Although congenital cholesteatomas (according to Derlacki und Clemis^20^; *n* = 14) were more extensive at the first operation (*p* = 0.006), they were not associated with a higher risk of residual disease in the second surgery when compared to all cases of acquired cholesteatoma (*n* = 94; *p* = 0.584), as well as compared to acquired cholesteatoma in children (*n* = 31; *p* = 0.785). Residual rates were 64.3% for congenital cholesteatomas, 56.5% for all cases of acquired cholesteatoma and 58.1% for acquired cholesteatoma in children.

To assess the size of cholesteatomas, the middle ear was divided into 6 compartments (hypotympanum, mesotympanum, sinus tympani, attic, antrum, mastoid). Additionally, growth toward the Eustachian tube, petrous apex, and the chorda-facial angle was recorded. Involvement of the 110 cholesteatomas during first surgery was the following: attic 91.8%, antrum 60.0%, mesotympanum 51.8%, sinus tympani 45.5%, mastoid 41.8%, hypotympanum 29.1%, growth toward Eustachian tube 12.7%, facial angle 3.6%, petrous apex 2.7%. In 17 patients only one compartment was involved, in 23 patients 2 compartments, in 25 patients 3 compartments, in 24 patients 4 compartments, in 8 patients 5 compartments and in 13 patients 6 compartments. Cholesteatoma growth from the attic and mesotympanum (except sinus tympani) to the sinus tympani as well as to the antrum or the mastoid was identified as a risk factor for residual cholesteatoma. Cholesteatoma growth to the sinus tympani leads to a fourfold increased risk of residual disease when compared to cholesteatomas which were confined to the attic and mesotympanum (Table 2). The difference was statistically significant with *p* = 0.042. Also patients with a cholesteatoma extension to the antrum or mastoid had a 3.5 fold higher risk for recurrence. This difference was statistically significant with *p* = 0.020 (Table 2).

**Table 2:** Rates of residual cholesteatoma depending on cholesteatoma extension to the attic and mesotympanum (except sinus tympani) alone, attic and sinus tympani or attic and antrum in initial surgery.

Extension	Patients without residual cholesteatoma	Patients with residual cholesteatoma
Attic/mesotympanum	18 (66.7%)	9 (33.3%)
Attic + sinus tympani	6 (35.3%)	11 (64.7%)
Attic + antrum	12 (36.4%)	21 (63.6%)

The columns show the absolute and relative number of patients with and without residual cholesteatoma depending on the cholesteatoma extension from the attic to the sinus tympani or the antrum in the initial surgery.

An erosion of ossicles occurred in the majority of patients. Neither the sole erosion of the incus (*p* = 0.232), nor the erosion of more than one ossicle were statistically associated with a higher risk of residual cholesteatoma. The validity, nevertheless, is diminished because of the low number of patients with an unharmed incus (*n* = 10).

We differentiated between 5 techniques of cholesteatoma removal within this study: (1) CWD, (2) CWD with reconstruction of the previously removed parts of the posterior auditory canal wall and transmeatal approach (CWD + R 1 way), (3) CWD with reconstruction of the previously removed parts of the posterior auditory canal wall and combined transmeatal–transmastoidal approach (CWD + R 2 ways), (4) CWU with transmeatal approach (CWU 1 way) and (5) CWU with combined transmeatal–transmastoidal approach (CWU 2 ways). Of course, the surgical technique depends on the size and location of the cholesteatoma. 14 cholesteatomas were restricted to the attic. Because of the low number of patients with a small cholesteatoma, no statistical difference was found between the chosen surgical techniques. In extended epitympanic cholesteatomas (attic + antrum; *n* = 33) rates of residual disease were significantly lower for the CWD + R 2 ways-surgery (25.0%) when compared to all other techniques (76.0%) excluding CWD. This difference was statistically significant (*p* = 0.015). Considering only CWD + R surgeries for these cholesteatomas, patients with a 2 ways approach (*n* = 8) had a 16 fold lower recurrence risk (*p* = 0.006) when compared to patients with a 1 way approach (*n* = 19). Patients with an extended epitympanic or mesotympanic cholesteatoma (*n* = 76) growing toward the antrum and/or the sinus tympani had a fourfold reduced risk of residual cholesteatoma when operated with the CWD + R 2 ways approach (*n* = 17; 41.2% recurrence) compared to all other techniques except CWD (*n* = 59; 74.6% recurrence). This difference was statistically significant (*p* = 0.010). Again, considering only CWD + R for these cholesteatomas (*n* = 71), patients with a 2 ways approach (*n* = 17) had a fourfold lower risk of residual disease (*p* = 0.012) when compared to patients with a 1 way approach (*n* = 54). Seven patients with an extended epitympanic and mesotympanic cholesteatoma had a CWD surgery. The rate of residual disease of 42.0% is comparable to the rate of CWD + R 2 ways. Only two patients had a cholesteatoma restricted to the mesotympanum. They were both operated in a transmeatal approach (1 × CWD + R 1 way, 1 × CWU 1 way). Both suffered from residual disease.

Considering all 110 cases there was no difference in the rate of residual disease for patients only underwent an atticotomy or patients having had both an atticotomy and an antrotomy. Understandably, a pure atticotomy is not sufficient for the removal of an extended cholesteatoma. If only cholesteatomas restricted to the attic are considered, the rate of residual cholesteatoma seems to be higher for patients who had only received an atticotomy (44.4%) when compared to those who had received an additional antrotomy (11.1%). Nevertheless, this difference was not statistically significant with *p* = 0.193. The resection of the chorda tympani (*n* = 27/110) was associated with an increased risk for residual disease (*p* = 0.038). However, in a multivariate analysis the resection of the chorda showed a clear correlation to the extension of the cholesteatoma and was therefore not identified as an independent risk factor.

107 patients underwent a tympanoplasty in the first operation. No type of tympanoplasty was identified as a statistically significant risk factor. The use of autologous material for the reconstruction of the ossicles tendentially showed a higher rate of residual disease when compared to tympanoplastic techniques without replantation of osseous tissue from the diseased ear. However, the difference was not statistically significant (*p* = 0.127). For the reconstruction of the tympanic membrane, perichondrium (*n* = 80), fascia (*n* = 25) and/or cartilage (*n* = 49) were used. None of these three tissues was associated with an increased risk for residual cholesteatoma in the second operation.

### Risk factors in between first and second surgery

A retraction of the anterior or posterior superior quadrant before the second surgery was identified as a statistically significant risk factor for residual cholesteatoma with *p* = 0.019 (OR = 4.4). Also a perforation of the anterior or posterior superior quadrant was associated with a higher rate of residual disease (*p* = 0.042), but was not an independent risk factor in a multivariate analysis as it depended on retraction. Patients with otorrhea tendentially suffered from residual cholesteatoma more often than patients without otorrhea (68.4% vs. 50.0%). However, the difference was not statistically significant (*p* = 0.066). Additionally, by multivariate analysis, otorrhea was not identified as an independent risk factor as it depended on retraction and perforation. Also the hearing loss as verified in pure tone audiometry after initial cholesteatoma removal was not associated with a higher rate of residual disease (*p* = 0.399). Nevertheless, a flattened, Type B tympanogram (*n* = 70/87 patients, missing patients had no tympanogram prior to the second surgery) was associated with a fivefold higher risk of residual cholesteatoma (67.1%) when compared with a regular Type A tympanogram (*n* = 17, recurrence rate 29.4%). The difference was statistically highly significant (*p* = 0.004) and the flattened tympanogram could be identified as an independent risk factor in a multivariate analysis, namely including retraction and perforation.

### Score for risk assessment regarding residual cholesteatoma

On the basis of parameters which were significantly associated with a residual cholesteatoma, a score was elaborated in order to facilitate the decision for or against a second-look surgery after cholesteatoma removal (Table 3). The parameters integrated into the score were the postoperative retraction or perforation of the tympanic membrane as well as the quality of the tympanogram and the intraoperative extension of the cholesteatoma to the sinus tympani and/or the antrum. In total 0–6 points could be assigned. Patients with 0–2 points were classified into Group A (“low risk”) and patients with 3–6 points into Group B (“high risk”). From the 48 patients allocated to Group A only 17 had a cholesteatoma during second-look surgery (35.4%), while in 35 of the 39 patients in Group B a residual cholesteatoma was found (89.7%). Thus, patients from Group B had a 26 fold higher risk for residual cholesteatoma. This difference was statistically highly significant (*p* = 0.001). Only 87 of the 110 investigated cases could be analyzed, as a tympanogram prior to the second-look operation was not available in the remaining 23 cases. Sensitivity and specificity regarding the prediction of a residual cholesteatoma using the score were calculated with 67.0% and 89.0%, respectively, while the positive and negative predictive value accounted to 90.0% and 65.0%, respectively.

**Table 3:** Score for risk assessment regarding residual cholesteatoma.

Parameter	Points	
	Yes	No
Postoperative perforation and/or retraction of the superior quadrants of the tympanic membrane	3	0
Flattened postoperative tympanogram	1	0
Intraoperative extension to sinus tympani	1	0
Intraoperative extension to antrum	1	0

The table shows relevant intra- and postoperative parameters for residual cholesteatoma. The points are added to a score.

## Discussion

The only effective therapy for cholesteatoma is complete surgical removal. However, involuntarily remaining cholesteatoma tissue leads to an early recurrence of the disease.^18^ Different risk factors have been described in the literature. Unfortunately, studies are hard to compare as they were conducted in different countries with different surgical standards and surgical concepts and partially have observation periods reaching back to the 1970ies. Furthermore, most previous studies only investigated infantile cholesteatomas. Therefore, our current retrospective study included children as well as adults in order to identify risk factors for residual cholesteatoma irrespectively of the patients’ age. Risk factors were subdivided into preoperative, intraoperative, and postoperative parameters referring to the first surgery.

Spilsbury et al. has recently documented on an impressively high number of 45,980 children that a history of ventilation tubes was associated with a higher risk of initial cholesteatoma formation.^4^ This is in line with the relatively high number of patients (about one third) with previous ventilation tube insertion in our current study. However, we could not identify previous ventilation tubes as a significant risk factor for residual disease. The decision in favor or against the insertion of ventilation tubes and the indication of a semi-prophylactic tympanoplasty due to an adhesive otitis media vary considerably among different countries and even among different surgeons, making a comparison of different studies difficult.^21^ Additionally, our study involved patients independent of their age. Consequently, we also analyzed many cholesteatomas in adults where usually the incidence of a previous ventilation tube insertion is lower than in an infantile cohort. In contrast, previous adenoidectomy came out as a statistically significant risk factor for residual cholesteatoma in our current study. A possible explanation is the risk of scar formation after adenoid removal which could lead to a chronic ventilation disorder and consequently to an atrophy of the tympanic membrane, an adhesive otitis media, and more difficult surgical conditions in initial surgery.^22^ Furthermore, adenoidectomy is usually performed in cases of chronic ventilation problems with recurrent otitis and formation of retraction pockets thereby presumably reflecting the increased risk of cholesteatoma formation in already compromised middle ears. The intraoperative extension of the cholesteatoma to the sinus tympani, the antrum, and the mastoid was associated with a statistically higher risk of residual disease in accordance to the literature.^6^, ^8^, ^10^, ^11^, ^13^, ^14^ This seems to be self-explanatory as the larger a cholesteatoma is, the greater is the risk of residual cholesteatoma tissue. Furthermore, the visualization of the middle ear compartments affected by the cholesteatoma can be insufficient in advanced disease. The hypothesis that the erosion of ossicles is a risk factor for recurrence,^6^, ^10^, ^11^, ^12^, ^15^ can neither be approved nor disproved by our data as there were few patients with a completely intact ossicular chain. This phenomenon is due to the specific patient population of a tertiary-referral ENT hospital where predominantly patients with an extended cholesteatoma were treated. The different techniques of cholesteatoma removal have also been discussed as being associated with different rates of residual disease. We found similar rates of residual cholesteatoma in cases of CWD surgery (33%) and CWD + R 2 ways surgery (41%), while all other surgical techniques were associated with higher rates of residual disease. Recently, many authors have stated that the CWU surgery is not a relevant risk factor for residual cholesteatoma.^6^, ^10^, ^11^, ^12^, ^13^, ^23^ In the present study, however, the CWD + R 2 ways-approach was clearly superior to the CWU technique in avoiding residual cholesteatoma, except in cases of small epitympanic cholesteatomas. These small cholesteatomas could be sufficiently resected without partial removal of the external ear canal wall. However, the number of patients with limited cholesteatoma growth in our patient population was too small to allow a valid statistical analysis. All in all, cholesteatoma removal should be adapted to its extension and a rather aggressive removal seems favorable regarding residual disease. On the one hand, a consecutive reconstruction of the previously removed parts of the posterior wall of the auditory canal in CWD + R 2 ways approaches does not seem to be disadvantageous regarding residual cholesteatoma, although there is in principle an imminent risk of burying residual epithelia behind the inserted cartilage. On the other hand, it restores the natural anatomy of the external auditory canal, thus avoiding the problems coming along with extensive postoperative cavities. When compared to the rates of residual disease in literature, which are usually indicated as lowest with 4–25% after CWD surgery, with 9–70% after CWU surgery, and with 10–27% after reconstruction of the posterior auditory canal wall, if named separately at all,^7^, ^8^, ^9^, ^10^, ^12^, ^13^, ^15^, ^16^, ^24^, ^25^ the rates of residual cholesteatoma in this study were generally higher. This can be explained by the high number of extended cholesteatomas in our study, reflected by the high rate of patients undergoing CWD + R surgery (about 60.0%) and the low number of patients receiving a CWU operation (about 30%). Furthermore, this might also be an effect of the selected study design. In most studies, patients who are not operated for a second time are considered as free of cholesteatoma while in this study we evaluated only patients who were effectively operated for a second time, due to either a recommendation for a second-look surgery or a suspicion of residual disease during follow-up, thus already representing a group of high risk patients. Another interesting approach for consideration is endoscopic cholesteatoma removal. As for now, there seem to be promising results regarding residual or recurrent pathology for this minimally invasive treatment.^26^

The type of tympanoplasty after cholesteatoma removal did not have any influence on the rate of residual cholesteatoma. For ossicular chain reconstruction the rates of residual disease were slightly higher for autologous implants when compared to alloplastic material. With respect to the comparable hearing results^27^ the re-implantation of auto-ossicles after cholesteatoma removal should therefore be performed restrictively.

Both the postoperative retraction and perforation of the upper quadrants of the reconstructed tympanic membrane were identified as risk factors for residual disease, which is in accordance to the literature.^13^, ^28^ Otorrhea was often present in relapses,^13^, ^28^ but usually together with a re-perforation of the eardrum and not as an isolated symptom. Although progressive postoperative hearing loss was identified as an independent risk factor in the present study, it is self-evident that hearing impairment should result in an accurate inspection of the eardrum. Importantly, stable postoperative hearing and especially a Type A tympanogram indicated a reduced probability of residual cholesteatoma, presumably reflecting normal middle ear ventilation.

In the present study children and adults had comparable rates of residual cholesteatoma (60.0% vs. 55.4%). The current literature has not been able to determine to date if age is really an independent risk factor for residual cholesteatoma.^13^, ^17^, ^29^ Histopathologically, there is no difference between cholesteatomas in adults and children,^30^ but cholesteatomas are considered to have a larger extension^17^ and to be more often super-infected in children, thus mimicking aggressive growth.^31^ Despite the often delayed diagnosis, we did not identify congenital cholesteatomas as a statistically significant risk factor for residual disease in this study. This is in accordance with the recent literature^8^, ^25^, ^32^ and might be due to the fact that children with congenital cholesteatomas usually do not have chronic ventilation problems and thus are not affected by an atrophy of the tympanic membrane and the associated difficult surgical conditions at initial surgery. In recent years, with advances in modern imaging, the second-look surgery is more and more a topic of controversial discussion.^33^ In several studies imaging was compared with intraoperative findings. As a result, both initially diagnosed cholesteatomas and residual disease can be detected with the aid of non-echo planar diffusion weighted magnetic resonance imaging (and computed tomography) with high sensitivity and specificity.^19^, ^34^, ^35^ In order to avoid financial burdens caused by the expense of modern sectional image analysis, it is crucial to identify parameters which suggest high rates of residual disease in order to filter out those patients who really need a follow-up with imaging and/or second-look surgery. In this context we developed a score by which residual disease can be predicted with relatively high sensitivity and specificity. The score, including the intraoperative extension of the cholesteatoma to the sinus tympani and/or the antrum, the development of a postoperative retraction or perforation of the reconstructed tympanic membrane, and the type of postoperative tympanogram, provides the opportunity to assign each patient to a group with low or high risk of residual cholesteatoma. On the one hand, the score is quite simple, allowing unpretentious application in clinical routine. On the other hand, it highlights the great clinical importance of post-operative retraction pocket formation as a risk factor for residual cholesteatoma, as patients with post-operative retractions alone are statistically prone to residual disease. We suggest that “high-risk” patients, as identified by the score, should undergo sectional imaging and, in uncertain cases, consecutive second-look surgery.

## Conclusion

The only treatment of cholesteatomas remains surgical removal, mostly combined with a consecutive reconstruction of the hearing apparatus. However, cholesteatomas tend to relapse. This study aimed at identifying anamnestic, clinical, and surgery-related risk factors for residual disease. Cholesteatoma extension to the sinus tympani and/or the antrum and a retraction or perforation of the reconstructed tympanic membrane were strongly associated with residual cholesteatoma, while a regular type A tympanogram makes residual disease unlikely. For extended cholesteatomas the CWD + R 2 ways approach seems to be safe without the disadvantages of an extensive postoperative cavity, thus being more convenient for the patients concerned. We developed a score including the intraoperative extension of the cholesteatoma, the type of the postoperative tympanogram and the postoperative retraction and perforation of the tympanic membrane to assess the risk of residual cholesteatoma. This score might be more objective than the personal recommendation of a surgeon after initial cholesteatoma removal and should be used to filter out high-risk patients needing further diagnosis as well as regular clinical follow-up.

## Conflicts of interest

The authors declare no conflicts of interest.
